# Investigation of a synonymous mutation in *Btk* in a patient with agammaglobulinemia: A case report

**DOI:** 10.1002/iid3.1049

**Published:** 2023-10-20

**Authors:** Cindy Srinivasan, Afshin Shameli, Bruce Ritchie, Adil Adatia

**Affiliations:** ^1^ Student, Department of Medicine University of Alberta Edmonton Alberta Canada; ^2^ Alberta Precision Laboratories, Calgary, Alberta, and Department of Laboratory Medicine and Pathology University of Washington Seattle Washington USA; ^3^ Division of Hematology University of Alberta Edmonton Alberta Canada; ^4^ Department of Medicine, Division of Pulmonary Medicine University of Alberta Edmonton Alberta Canada

**Keywords:** antibody deficiency, case report, inborn errors of immunity, primary immunodeficiency, X‐linked agammaglobulinemia

## Abstract

**Background:**

X‐linked agammaglobulinemia (XLA) is the most common form of agammaglobulinemia and is caused by mutations in Btk, which encodes Bruton tyrosine kinase (BTK).

**Case Description:**

We describe a 36‐year‐old male who presented as an infant with hypogammaglobulinemia and sinopulmonary infections and was initially diagnosed with common variable immunodeficiency. Genetic testing showed he was hemizygous for Btk c.240G > A. This synonymous variant affecting the last nucleotide of exon 3 leads to aberrant splicing of most but not all mRNA transcripts.

**Conclusion:**

We demonstrated reduced BTK protein expression confirming the pathogenicity of the variant and related our findings to genotype‐phenotype relationship studies ina XLA caused by synonymous mutations.

## Graphical abstract

We describe a case of X‐linked agammaglobulinemia (XLA) caused by the synonymous mutation Btk c.240 G > A, which results in aberrant splicing (and presumed nonsense mediated decay), but also leads to low level residual wild‐type BTK messenger RNA. We demonstrate detectable but significantly reduced BTK expression the patient's monocytes and relate this result and the patient's clinical course to the open question of genotype‐phenotype relationships in XLA.

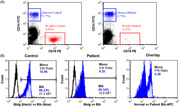

## 1 INTRODUCTION

Bruton tyrosine kinase (BTK) is a cytoplasmic tyrosine kinase that is critical for the development of B cells. Loss of BTK enzyme function causes X‐linked agammaglobulinemia (XLA) due to B cell developmental arrest at the pro‐ to pre‐B cell stage in the bone marrow, and consequently absent circulating B cells and profoundly decreased serum antibody concentrations.^1^, ^2^ Patients characteristically present with sinopulmonary and gastrointestinal infections in infancy after 6 months of age when maternal IgG is no longer present. Patients are treated with life‐long immunoglobulin replacement, but many develop progressive obstructive lung disease over time despite therapy.^3^ Herein, we describe a case of a patient affected by a synonymous splice‐site mutation in*Btk* resulting in a mild phenotype of XLA.

## 2 METHODS

### 2.1 Clinical data

A 36‐year‐old male presented to our immunodeficiency center for reevaluation and transfer of care. As an infant, he had multiple emergency department visits for otitis media and for lower respiratory symptoms that were attributed to reactive airways. He was found to have hypogammaglobulinemia at age 3 after he was hospitalized for pneumonia complicated by febrile seizures. He was diagnosed with common variable immunodeficiency (CVID) and started on intravenous immunoglobulin replacement at a dose of 0.6 g/kg every 8 weeks. After starting treatment, his burden of infections dramatically decreased, and throughout his childhood and adulthood he required antibiotics for sinopulmonary infections less than once per year. There was no history of central nervous system, gastrointestinal, or joint infection, and he did not have any documented infections with opportunistic organisms. He did not have any chronic respiratory symptoms to suggest bronchiectasis. Since age 30 years, he has been receiving intravenous immunoglobulin 0.6 g/kg every 6 weeks; this extended dosing interval was used because of patient preference and maintained IgG trough concentrations of 6 g/L. The patient has two asymptomatic sons.

### 2.2 Diagnostic procedures

Sequencing and copy number variant analysis of a panel of 642 genes including *Btk* was performed at Blueprint Genetics. BTK protein expression was measured by flow cytometry at Alberta Precision Laboratories (Calgary, AB). Fifty microliters of the peripheral blood sample was added to each sample and incubated with 5 µL of the Fixative Reagent from PerFix‐nc kit (Beckman Coulter, Brea, CA) for 15 min at room temperature. This was followed by the addition of 300 µl of the Permeabilizing Reagent. Antibodies against surface markers and BTK (or isotype control) were then added and incubated for 60 min in the dark at room temperature. The mixture was washed with Phosphate Buffered Saline (PBS)/2% Bovine Serum Albumin, followed by a second wash with PerFix‐nc buffer. The pellet was resuspended in PerFix‐nc buffer 3, followed by acquisition on a Navios flow cytometer (Beckman Coulter). Antibodies against following markers were used: CD45 (clone HI30)‐BV510, CD14 (clone 63D3)‐Alexa Fluor 488 (BioLegend, Inc.), CD19 (clone 89B)‐RD1 (Beckman Coulter), BTK (clone 53/BTK)‐Alexa Fluor 647 and Mouse IgG1 isotype control (clone MOPC‐21)‐Alexa Fluor 647 (BD Biosciences).

## 3 RESULTS

**Figure 1:**
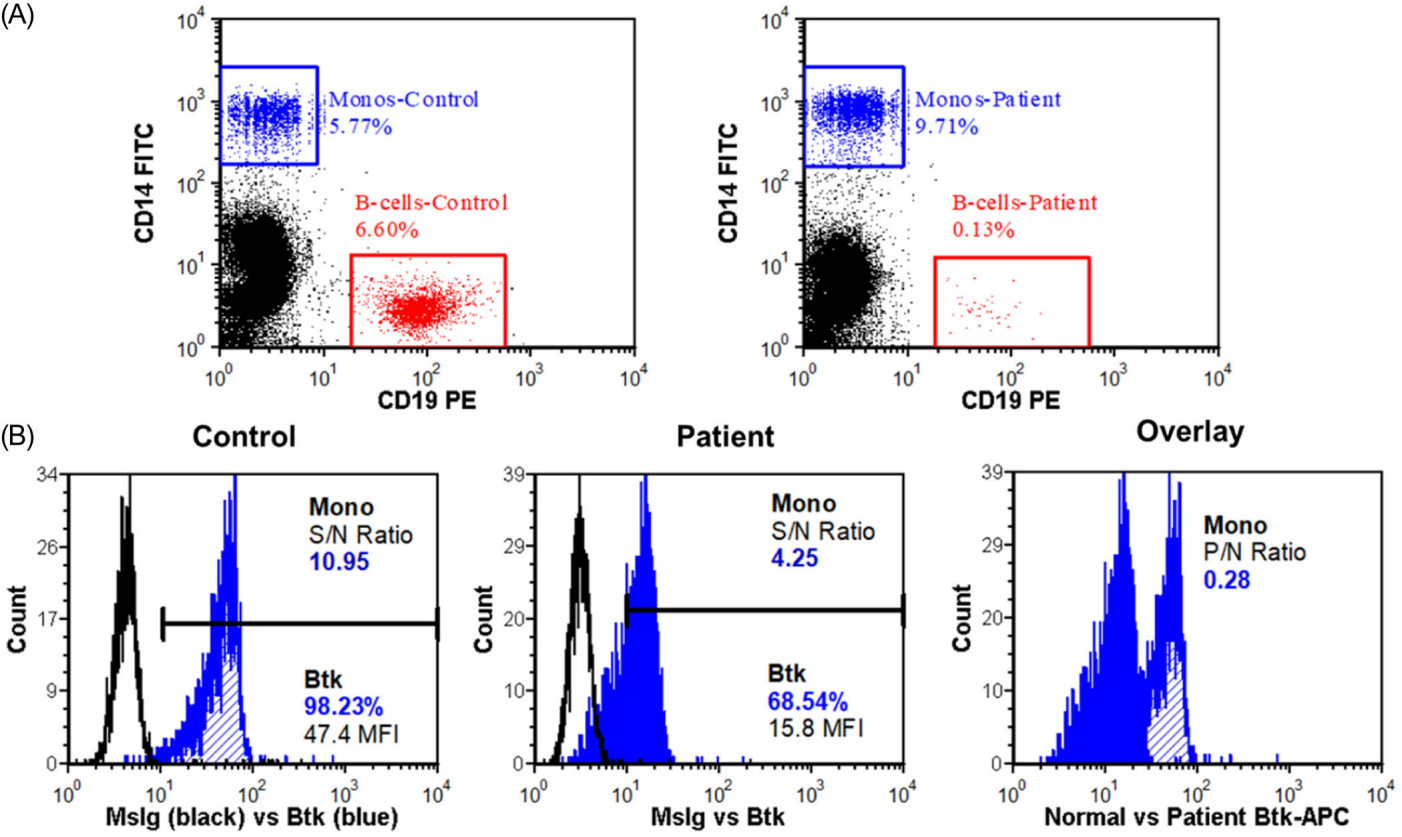
(A) B cell enumeration for a healthy control (left) and patient (right) peripheral blood samples. The patient's B cells constituted only 0.13% of total lymphocytes. (B) Flow cytometric measurement of BTK expression in monocytes of the healthy control (left), patient (middle), and both overlayed (right). BTK, Bruton tyrosine kinase.

His laboratory and pulmonary function results are shown in Table 1. His IgG was 6.47 g/L (on treatment), his IgA was normal at 1.80 g/L, and his IgM was undetectable. His lymphocyte subsets were remarkable for nearly absent B cells (Figure 1A). His T cell counts and mitogen responses to phytohemagglutinin and concanavalin A were normal. His spirometry, lung volumes, and diffusion capacity for carbon monoxide were also normal.

**Table 1:** Patient laboratory and pulmonary function results.

Investigations	Results (Reference Range)
Lymphocyte Subsets (x10^9^/L)	
Total Lymphocytes	1.110 (1.0−4.8)
CD4+ T cells	0.470 (0.500−2.00)
CD8+ T cells	0.310 (0.200−1.200)
B cells	0.000 (0.064−0.820)
NK cells	330 (0.100−1.200)
Mitogen Proliferation Indices	
PHA	2.14 (≥1.40)
ConA	3.58 (≥1.40)
Immunoglobulins (g/L)	
IgG	6.47 (6.90−16.0)
IgA	1.80 (0.70−4.00)
IgM	<0.30 (0.60−3.00)
Pulmonary function (units, (% predicted))	
FEV1 (L)	5.14 (110)
FVC (L)	5.91 (103)
TLC (L)	7.21 (93)
DLCO (mL/mmHg/min)	37.3 (104)

Abbreviations: ConA, concanavalin A; DLCO, diffusing capacity for carbon monoxide; FEV1, forced expiratory volume in 1 s; FVC, forced vital capacity; TLC, total lung capacity; PHA, phytohemagglutinin.

Gene sequencing identified the variant *Btk* c.240 G > A, a synonymous missense mutation affecting the last nucleotide of exon 3. *In silico* analysis showed that the variant disrupted a donor splice site at the interface between exon 3 and intron 4 and was predicted to be damaging.^4^ The variant is absent from the gnomAD reference database.

Flow cytometry with intracellular staining of BTK in monocytes showed severely reduced but detectable BTK expression with a mean fluorescence intensity ratio (patient:control) of 0.28, consistent with a diagnosis of XLA.

## 4 DISCUSSION

We present a patient with XLA caused by the synonymous mutation *Btk* c.240 G > A. This mutation has been reported in 3 unrelated patients with agammaglobulinemia.^5^, ^6^, ^7^ Its pathogenicity has been inferred by messenger RNA (mRNA) sequencing performed by Noordzij et al.^6^ showing that this variant resulted in the loss of a donor‐acceptor site leading to an out‐of‐frame insertion of 106 nucleotides from intron 3, which introduced a premature stop codon. This aberrant transcript is expected to undergo nonsense‐mediated decay. The same group found that 3% of*Btk* transcripts in the patient were wild‐type, but they were unable to detect expression of BTK by western blot. Here we show using flow cytometry with a modern anti‐BTK antibody that, as predicted, this variant does result in detectable but significantly reduced BTK protein expression at 28% of control. Interestingly, this is much higher than might be anticipated if there was less than 5% of wild‐type mRNA expression, which may be related to some degree of increased survival or proliferation of monocytes with greater BTK expression.

Some authors have posited that synonymous mutations that permit some residual wild‐type *Btk* transcription result in a milder phenotype.^6^ The present patient presented as an infant with pneumonia, consistent with the fully penetrant phenotype. However, he has had no significant infections and maintains normal lung function while treated with (suboptimal) immunoglobulin therapy, and he has no autoimmune complications. We thus consider him to have milder disease.

Predicting disease severity from genotype in XLA is highly desirable as a subset of patients develop early obstructive lung disease and bronchiectasis,^8^ and these patients may benefit from enhanced monitoring with more frequent pulmonary function studies and chest computed tomography, and perhaps higher doses of immunoglobulin before the onset of lung disease.^9^

Identification of clear genotype‐phenotype correlations in XLA in larger cohorts have remained elusive, however. Earlier studies used surrogate markers such as age of first presentation, higher percentages of circulating mature B cells, and presence of preserved IgA/IgM production^9^, ^10^, ^11^ to infer disease severity. More recently, Lougaris et al.^3^ described a large cohort with long‐term follow up of clinically important outcomes and no clear relationships were identified,^3^ but subgroup analysis of those with synonymous splice site mutations resulting in residual wild‐type BTK expression was not possible from the available data.

In conclusion, we present a patient initally diagnosed with CVID whom upon further investigation was found to have XLA. We show that the synonyous *Btk* c.240 G > A mutation results in residual BTK expression and postulate that this is the reason for his favorable disease course. Further long‐term studies of patient‐important outcomes (e.g., infection frequency and development of chronic lung disease), and different types of splice site mutations may yet identify useful genotype‐phenotype correlations in a subset of patients.

## AUTHOR CONTRIBUTIONS

**Cindy Srinivasan**: Data curation; visualization; writing—original draft. **Afshin Shameli**: Formal analysis; methodology; resources; validation; writing—original draft. **Bruce Ritchie**: Conceptualization; methodology; resources; writing—review and editing. **Adil Adatia**: Conceptualization; formal analysis; project administration; supervision; writing—original draft; writing—review and editing.

## CONFLICTS OF INTEREST STATEMENT

A. A. reports grants from Ionis and Astria, and conference travel support and honoraria from Takeda, CSL‐Behring, and BioCryst, outside of the submitted work. B. R. reports grants from Ionis, CSL‐Behring, Takeda, OctaPharma, Alynlam, and Mitsubishi Tanbe and travel support from BioCryst, outside of the submitted work. The remaining authors declare no conflict of interest.

## ETHICS STATEMENT

Written informed consent has been obtained from the patient to publish this paper.

## Data Availability

Data available on request due to privacy/ethical restrictions.
